# Paediatric major incident triage: UK military tool offers best performance in predicting the need for time-critical major surgical and resuscitative intervention

**DOI:** 10.1016/j.eclinm.2021.101100

**Published:** 2021-08-23

**Authors:** Nabeela S. Malik, Saisakul Chernbumroong, Yuanwei Xu, James Vassallo, Justine Lee, Christopher G. Moran, Tina Newton, G. Suren Arul, Janet M. Lord, Antonio Belli, Damian Keene, Mark Foster, Timothy Hodgetts, Douglas M. Bowley, Georgios V. Gkoutos

**Affiliations:** a NIHR Surgical Reconstruction and Microbiological Research Centre (SRMRC), Heritage Building, Queen Elizabeth Hospital, Mindelsohn Way, Edgbaston, Birmingham B15 2TH, United Kingdom; b Institute of Inflammation and Ageing, University of Birmingham, Birmingham B15 2TT, United Kingdom; c 212 (Yorkshire) Field Hospital, Endcliffe Hall, Endcliffe Vale Road, Sheffield S10 3EU, United Kingdom; d Institute of Cancer and Genomic Sciences, University of Birmingham, Birmingham B15 2TT, United Kingdom; e Academic Department of Military Emergency Medicine, Royal Centre for Defence Medicine, Mindelsohn Way, Edgbaston, Birmingham B15 2WB, United Kingdom; f University Hospitals Birmingham, Mindelsohn Way, Edgbaston, Birmingham B15 2WB, United Kingdom; g NHS England London, Skipton House, 80 London Road, London SE1 6LH, United Kingdom; h Nottingham University Hospitals NHS Trust, Derby Road, Nottingham NG7 2UH, United Kingdom; i Birmingham Children's Hospital, Steelhouse Lane, Birmingham B4 6NH, United Kingdom; j Academic Department of Military Surgery & Trauma, Royal Centre for Defence Medicine, Mindelsohn Way, Edgbaston, Birmingham B15 2WB, United Kingdom; k Headquarters Defence Medical Services, Coltman House, Lichfield WS14 9PY, United Kingdom; l Institute of Translational Medicine, University Hospitals Birmingham NHS Foundation Trust, Birmingham B15 2TT, United Kingdom; m MRC Health Data Research UK (HDR UK), Midlands Site, Birmingham B15 2TT, United Kingdom

## Abstract

**Background:**

Children are frequently injured during major incidents (MI), including terrorist attacks, conflict and natural disasters. Triage facilitates healthcare resource allocation in order to maximise overall survival. A critical function of MI triage tools is to identify patients needing time-critical major resuscitative and surgical intervention (Priority 1 (P1) status). This study compares the performance of 11 MI triage tools in predicting P1 status in children from the UK Trauma Audit and Research Network (TARN) registry.

**Methods:**

Patients aged <16 years within TARN (January 2008-December 2017) were included. 11 triage tools were applied to patients’ first recorded pre-hospital physiology. Patients were retrospectively assigned triage categories (P1, P2, P3, Expectant or Dead) using predefined intervention-based criteria. Tools’ performance in <16s were evaluated within four-yearly age subgroups, comparing tool-predicted and intervention-based priority status.

**Findings:**

Amongst 4962 patients, mortality was 1.1% (*n* = 53); median Injury Severity Score (ISS) was 9 (IQR 9–16). Blunt injuries predominated (94.4%). 1343 (27.1%) met intervention-based criteria for P1, exhibiting greater intensive care requirement (60.2% vs. 8.5%, *p* < 0.01) and ISS (median 17 vs 9, *p* < 0.01) compared with P2 patients. The Battlefield Casualty Drills (BCD) Triage Sieve had greatest sensitivity (75.7%) in predicting P1 status in children <16 years, demonstrating a 38.4–49.8% improvement across all subgroups of children <12 years compared with the UK's current Paediatric Triage Tape (PTT). JumpSTART demonstrated low sensitivity in predicting P1 status in 4 to 8 year olds (35.5%) and 0 to 4 year olds (28.5%), and was outperformed by its adult counterpart START (60.6% and 59.6%).

**Interpretation:**

The BCD Triage Sieve had greatest sensitivity in predicting P1 status in this paediatric trauma registry population: we recommend it replaces the PTT in UK practice. Users of JumpSTART may consider alternative tools. We recommend Lerner's triage category definitions when conducting MI evaluations.

## 1 Introduction

Children are often injured during major incidents (MI) including natural disasters, conflict and terrorist attacks, where their immediate needs exceed the resources available to treat them [1–4]. For example, following the 2017 Manchester Arena Bombing, children constituted 44/153 (29%) casualties attending various Emergency Departments (ED) [5] and 7/22 (32%) of those killed [2]. During MI, resources are best directed towards maximising overall survival amongst those affected. Selection of a triage tool for use at scene is an important aspect of disaster planning, enabling patients to be prioritised for treatment and onward transfer, particularly those in need of immediate life-saving intervention[1,3,4]. Children display age-dependent normal vital signs and, thus adult tools may assign an incorrect triage category when applied in children [6]. There is a natural tendency for first responders to assign a higher triage category to children [6]; uninjured ambulatory children are often conveyed to hospital from MIs, as reported following the Fairchild and Columbine School massacres [4]. Incorrect triage of children may fail to identify those needing urgent intervention (under-triage); however, assigning P1 status to children who do not require time-critical treatment (over-triage) risks overwhelming dedicated paediatric resources at scene and in hospital, and potentially directing resources away from others who require intervention more urgently [7]. As such, objective assessment of injured children using an appropriate triage tool is crucial to maximising overall survival following a MI.

**Fig. 1:**
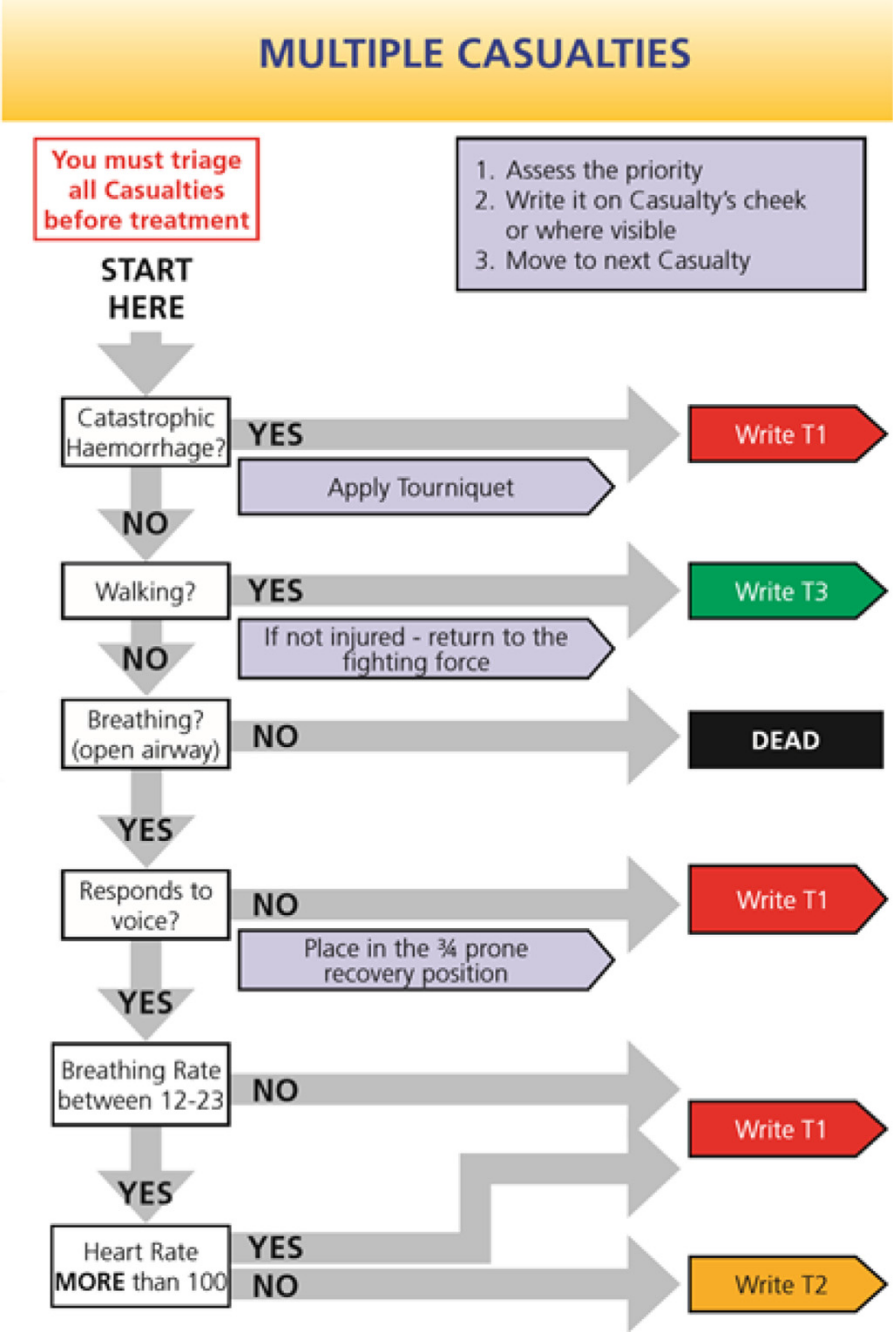
Battlefield Casualty Drills Triage Sieve (UK Military Primary Triage Tool).

The ideal MI triage tool is quick and simple to apply, with high sensitivity in identifying those for whom timely intervention is likely to alter overall outcomes (P1 patients) and an acceptably low rate of overtriage [8,9]. Two dedicated paediatric primary MI tools are in current use internationally. The Paediatric Triage Tape (PTT) (adapted from the adult MIMMS Triage Sieve in 1998) is applied to children <12 years in UK MIs [6]. PTT utilises physiological parameters relating to the height (or weight) of the child to determine the child's triage category. The US-based JumpSTART is a paediatric adaptation of the adult Simple Triage and Rapid Treatment (START) for use in children <8 years [10]. The Australian CareFlight triage tool has been applied in both adults and children, achieving good performance (AUC 0.852) in predicting mortality to discharge [11] and the need for intervention [12,13]. Although several other adult triage tools have been developed and are in use, their performance in children remains largely unvalidated [1]. These include the Major Incident Medical Management and Support (MIMMS) Triage Sieve [14], the Modified Physiological Triage Tool (MPTT) [15], its derivative MPTT-24 [15], the modified START (MSTART) [14], National Ambulance Resilience Unit (NARU) Triage Sieve [16] and the US-based Rapid Assessment of Mentation and Pulse (RAMP) [17]. The Battlefield Casualty Drills (BCD) Triage Sieve, used by British soldiers faced with multiple casualties, first appeared in 1998 [18], and was updated in 2018 in line with emerging evidence and changes in clinical practice (Fig. 1). The BCD Triage Sieve was recently identified as the most sensitive of multiple MI triage tools in an adult population [18].

Few studies exist to inform the choice of paediatric MI triage tools [1,11,19]. One challenge in interpreting existing studies lies with variation in age used to define a child [11,19,20]. Whilst the UK usually employs age <16 years as the cut-off for paediatric healthcare services, MI casualty distribution planning in some regions stipulates that children aged 12 to 16 years should be conveyed to adult facilities in order to preserve specialist paediatric services for the youngest patients. However no studies have examined tool performance in the 12 to 16 year old subgroup; in whom the NARU Triage Sieve would be applied (UK practice) [16]. Studies measuring triage tool performance have often focussed on predicting an Injury Severity Score (ISS) >15 as the end-point [13,20], despite a lack of correlation between ISS and requirement for medical resources [21]. There is growing consensus that the ability to predict requirement for urgent life-saving resuscitative and/or surgical intervention is the most meaningful measure of performance in MI triage tools [9,13,22]. Our primary aim was to determine which tool performs best in children (<16 years), in order to inform UK policy. A secondary aim was to analyse the performance of tools in subgroups of children by age, in order to determine the appropriateness of the age cut-offs applied by the paired adult and paediatric tools, namely the UK's Paediatric Triage Tape (<12 years) with the NARU Triage Sieve [6,16], and the US JumpSTART (below eight years) with START [10].

## 2 Methods

### 2.1 Overview of study design

This study utilises the physiology and outcomes of injured children within the UK national Trauma Audit and Research Network (TARN) as a surrogate for those injured in a MI. Two paediatric and nine adult triage tools have been applied to each patient's first recorded pre-hospital physiology. Patient records have been assessed to determine which triage category they would fulfil (P1, P2, Expectant or Dead) on the basis of required interventions, using pre-defined (Lerner's) criteria [9]; Priority 1 status was defined as patients requiring time-critical, major resuscitative and/or surgical intervention(s). Tool performance is reported against intervention-based Priority 1 status.

### 2.2 Study population

TARN co-ordinators capture pre-hospital, clinical management and outcome data from 169 trauma receiving hospitals in England and Wales, including all paediatric major trauma centres, constituting the largest trauma registry in Europe [23]. TARN includes injured patients attending hospital with length of stay over 48 h, intensive care admission and/or in-hospital death [23]. Pre-hospital deaths are excluded.

All patients aged <16 years submitted to TARN by hospitals in England and Wales between 1 January 2008 and 31 December 2017 were included. Those patients missing pre-hospital physiological data required for tool application (respiratory rate, heart rate, systolic blood pressure, Glasgow Coma Score (GCS), and GCS Motor Component) were excluded.

### 2.3 Application of triage tools

The BCD Triage Sieve (Fig. 1), CareFlight [14], JumpSTART [10], MIMMS Triage Sieve [14], MPTT [15], MPTT-24 [15], MSTART [14], NARU Triage Sieve[16], RAMP[17], START[14] and PTT[6] tools (see Table 1) were converted into computer code: these were verified by clinician co-authors and by application in an adult population [18]. Tool codes were applied to patients’ first recorded pre-hospital physiology (assuming that these preceded any intervention), to determine whether patients would be assigned P1 or non-P1 status, as per a recent adult study methodology [18].

**Table 1:** Summary of triage tool characteristics.

Tool	Description and geographical use	Tool components							
		1st step	2nd step	3rd step	4th step	5th step	6th step	7th step	Interventions permitted
Battlefield Casualty Drills (BCD) Triage Sieve	Current UK military tool for use in adults (introduced in 1998, revised 2018).	Catastrophic haemorrhage?	Walking?	Breathing?	Responds to voice?	Breathing rate between 12 and 23	Heart Rate more than 100	–	Apply tourniquet, open airway, place casualty in the ¾ prone recovery position
CareFlight	Australian tool used in adults and children (introduced in 2001).	Walks?	Obeys command?	Palpable radial pulse? OR Breathes with open airway?	–	–	–	–	Open airway
Jump Simple Triage and Rapid Treatment (JumpSTART)	United States, used in several states in children (introduced in 2001).	Able to walk?	Spontaneous breathing (check radial pulse if apnoeic)	Respiratory rate <15 or >45	Palpable pulse?	Neurological Assessment (AVPU)	–	–	Airway positioning, 5 rescue breaths if apnoeic
Major Incident Medical Management and Support (MIMMS) Triage Sieve	Former UK military adult triage tool (introduced in 1995).	Walking	Breathing	Respiratory rate <10 or ≥30	Capillary refill >2 s	–	–	–	Open airway
Modified Physiological Triage Tool (MPTT)	UK-based tool* modelled in a military cohort (described in 2017).	Walking?	Breathing?	Respiratory rate <12 or ≥22	Heart rate ≥100	GCS <14	–	–	–
Modified Physiological Triage Tool 24 (MPTT-24)	UK-based tool*, modification of MPTT (described in 2017).	Catastrophic Haemorrhage?	Walking?	Breathing?	Responds to voice	Respiratory rate <12 or ≥24	Heart rate ≥100	–	Apply tourniquet or haemostatic dressing
Modified Simple Triage and Rapid Treatment (MSTART)	United States, modification of START (described in 2006).	Able to walk?	Spontaneous breathing	Respiratory rate >30	Radial pulse absent	Obey commands	–	–	Position airway
National Ambulance and Resilience Unit (NARU) Triage Sieve	Current UK civilian adult tool, adapted from the MIMMS Triage Sieve (this version was introduced in 2013)	Catastrophic haemorrhage	Are they injured	Walking	Breathing	Unconscious	Respiratory rate <10 or ≥30	Pulse >120 or capillary refill >2 s	Apply tourniquet/haemostatic dressing, open airway, place in recovery position
Paediatric Triage Tape (PTT)	Current UK paediatric tool (<12 years) (adapted from MIMMS Triage Sieve in 1998).	Alert and moving all limbs (children <100 cm height) or Walking	Use tape to gauge child's length in order to determine which set of physiological values to compare the child against	Breathing?	Respiratory rate (height-specific threshold)	Capillary refill <2 s (use child's forehead)	Pulse rate (heigh-specific threshold)	–	Position airway
Rapid Assessment of Mentation and Pulse (RAMP)	United States, used by the Rocky Mountain Fire Department, Colorado (introduced in 2018).	Casualty without signs of obvious death	Casualty follows commands	Radial pulse present?	–	–	–	–	Control massive haemorrhage, open airway, chest decompression
Simple Triage and Rapid Treatment (START)	United States (introduced in 1983).	Able to walk?	Spontaneous breathing	Respiratory rate >30	Capillary refill >2 s	Obey commands	–	–	Position airway

Ledger: AVPU refers to the Alert, Voice, Pain, Unresponsive scale; GCS=Glasgow Coma Score. All tools described are applicable at the scene of a major incident (primary triage tools). *Has yet to undergo practical use or implementation studies. SALT and ASAV were not evaluated in this study as there were major limitations in applying these retrospectively. **SALT involves sorting according to the following: walk, wave/purposeful movement, still/obvious life threat; as well as the subjective judgements: “Minor injuries only?” and “Likely to survive given current resources?” ***ASAV includes the subjective judgement “Deadly injured?” and assessment of breathing status as follows: “airway obstructed, bradypnoea, apnoea, dyspnoea, tachypnoea (not obviously psychogenic) and cyanosis.”

Where tools employed parameters not recorded in TARN, approximations were made based on available information. TARN patients were assumed to be non-ambulatory. Those who underwent advanced airway interventions at scene were considered unable to breathe [24]. A respiratory rate below four breaths per minute was deemed undetectable by EMS personnel. No approximation for the term “catastrophic haemorrhage” (utilised by MPTT-24, BCD and NARU Triage Sieve) could be identified, hence this term was not applied. Children with a systolic blood pressure of ≥60 mmHg (<12 years) or ≥90 mmHg (≥12 years), were regarded as having a palpable radial pulse [25]. Patients with a GCS of ≤8 were deemed unconscious, those with a GCS <12 were deemed unresponsive to voice [26]. GCS Motor Score of six indicated ability to follow commands. For JumpSTART, a GCS Motor Score ≤3 was equated to “inappropriate response to painful stimulus (e.g. posturing) or unresponsive to noxious stimulus” [26].

Tool performance was measured in children <16 years and in subgroups based on age: 0 to <4 years (pre-school), 4 to <8 years, 8 to <12 years and age 12 to <16 years. These subgroups were selected in line with thresholds employed by the dedicated paediatric tools (PTT <12 years, JumpSTART <8 years).

### 2.4 Outcome measures

The primary outcome was the ability of triage tools to predict P1 status, defined as the need for any one or more of eight time-critical major resuscitative or surgical interventions (Table 2)[9]. Each patient was assigned a triage category (Dead, Expectant, Priority 1 [P1], Priority 2 [P2] or Priority 3[P3]) based on a pre-defined system utilising EMS and hospital-based interventions described by Lerner et al., using TARN terminology which best matched each criterion (see Supplementary Data Table 1). Since TARN does not include patients with chemical, biological, radiological, and nuclear injuries, criteria relevant to these mechanisms were not considered [9]. Two further paediatric-specific measures for “presented to ED with uncontrollable haemorrhage” were included: administration of a fluid bolus of 20 ml/kg within an hour of arrival in ED [22] and/or the requirement for blood products within an hour of ED arrival. In order to calculate the fluid bolus volume, weight was estimated using age as recorded by TARN and World Health Organisation male and female charts for infants up to 12 months [27], or the formula (age+2)x4 for children aged >12 months [28].

**Table 2:** Breakdown of time-critical major operative and resuscitative interventions constituting Priority 1 status.

Subcomponents of the Priority 1 triage category	n (%)
An advanced airway intervention (e·g· intubation, LMA, surgical airway) performed in the pre-hospital setting or within 4 h of arrival at hospital	808 (75·2%)
Arrived in the ED with uncontrolled haemorrhage	310 (28·9%)
Neurological, vascular, or haemorrhage-controlling surgery to the head, neck or torso performed within 4 h of arrival to hospital	169 (15·7%)
Chest tube placed within 2 h of arrival at hospital	70 (6·5%)
Limb-conserving surgery performed within 4 h of arrival at hospital on a limb that was found to be pulseless distal to the injury prior to surgery	23 (2·1%)
IV vasopressors administered within 2 h of arrival at hospital	6 (0·6%)
Patient who required EMS initiation of CPR (i·e· had a cardiac arrest) during transport, in the ED, or within 4 h of arrival at a hospital	1 (0·1%)
Escharotomy performed on a patient with burns within 2 h of arrival at a hospital	1 (0·1%)
Total number of P1 patients	1343 (100·0%)

Ledger: There is overlap between life-saving interventions (LSI): 73·2% (*n* = 1006) of P1 patients required one LSI, 21·8% (*n* = 299) required two LSI, and 5·0% (*n* = 69) required 3 or more LSI.

TARN records the timing of hospital arrival and each intervention, allowing incorporation of this into the time-critical definitions constituting P1 status. To assess the validity of Lerner's classification, patients within each category were compared by mortality, ICU admission, hospital LOS and ISS.

**Fig. 2:**
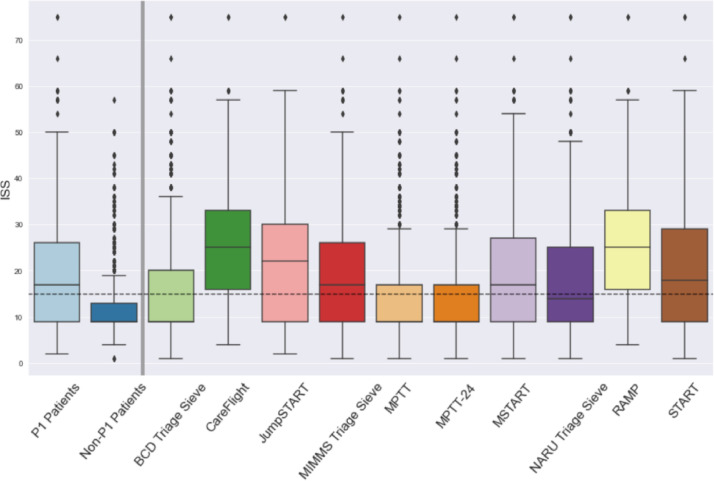
Distribution of ISS amongst tool P1 patients (patients aged ≤16 years) Ledger: ISS=Injury Severity Score. Dotted horizontal line denotes ISS 15. The upper whisker extends from the hinge to the largest value no further than 1•5 * IQR from the hinge; the lower whisker extends from the hinge to the smallest value, at most 1•5 * IQR of the hinge.

Secondary outcome measures included prediction of mortality and ISS>15 (see Supplementary data Table 2 and 3), and distribution of ISS amongst tool-assigned P1 patients (Fig. 2), which may provide further discriminative value and appreciation of tool characteristics.

**Table 3:** Summary of patient and injury characteristics.

Variable		
**Gender, *n* (%)**	Male	3447 (69·5%)
	Female	1515 (30·5%)
	Missing data	0 (0·0%)
**Injury Severity Score, median (IQR)**		9 (9, 17)
	Missing data	0
**Age (years)**	Median (IQR)	11·9 (8·0, 14·2)
	<16 years, n (%)	4962 (100·0%)
	0 to <4	467 (9·4%)
	4 to <8	768 (15·5%)
	8 to <12	1281 (25·80%)
	12 to <16	2446 (49·3%)
	Missing data	0 (0·0%)
**Outcome at discharge, *n* (%)**	Alive	4909 (98·9%)
	Dead	53 (1·1%)
	Missing data	0 (0·0%)
**Injury type, *n* (%)**	Blunt	4733 (95·4%)
	Penetrating	229 (4·6%)
	Missing data	0 (0·0%)
**Injury mechanism, *n* (%)**	Vehicle incident/collision	2459 (49·6%)
	Fall less than 2m	1187 (23·9%)
	Fall more than 2m	645 (13·0%)
	Blow(s)	327 (6·6%)
	Stabbing	130 (2·6%)
	Crush	42 (0·9%)
	Shooting	16 (0·3%)
	Blast	5 (0·1%)
	Burn	4 (0·1%)
	Other	147 (3·0%)
	Missing data	0 (0·0%)

### 2.5 Data processing and analyses

TARN data were received in SPSS Version 24·0 (Armonk NY: IBM Corp 2015) and processed using Python (Version 3.7.4) and R software (Version 3·6, R Core Team, New Zealand, 2000). Non-parametric data are presented as median and interquartile range; categorical data as frequency and percent. D'Agostino and Pearson's test was used to confirm the non-parametric nature of data distribution [29]. Differences between P1 and P2 patients as designated by Lerner's criteria [9] were compared using the Chi-squared test (mortality and ICU admission) and Mood's median test (ISS). Performance characteristics included sensitivity, specificity, under-triage (1-sensitivity) and over-triage (1-positive predictive value). Area Under the receiver operating Curve (AUC) was calculated using the trapezoidal rule [30]. 95% confidence intervals were calculated using the Wilson Score with continuity correction for binomial proportions, and DeLongs Algorithm for comparing AUC curves [31]. Included patients were compared to those excluded with respect to clinical and demographic characteristics. Fisher's exact test was used to compare categorical variables (gender, mortality and mode of injury (blunt and penetrating)). Continuous variables were compared using a Two-sample Kolmogorov-Smirnov Test. Differences in injury mechanism were estimated using the Chi-square test, where results were significant, post-hoc tests were performed to generate a *p* value. *P* values were adjusted using the Bonferroni correction. A value of *p* < 0·05 was considered statistically significant.

*Ethical approval:* The UK Health Research Authority Patient Information Advisory Group (Section 20) has granted ethical approval and waived the requirement for individual patient consent for research using anonymised TARN data.

*Role of Funding:* The funding source played no role in study design; in data collection, analysis or interpretation; in the writing of the report; or decision to submit the paper for publication.

## 3 Results

### 3.1 Characteristics of the study population

Of the 15,133 TARN patients identified, 10,171 (67·2%) patients were excluded due to incomplete pre-hospital physiological data (see Supplementary Data: Analysis of missing data), therefore, 4962 patients were included.

Patient and injury characteristics are presented in Table 3. Two thirds (69·5%) of patients were male, half (49·3%) were aged 12 to 16 years, whilst less than 10% (*n* = 467) were aged under four years. Mortality was 1·1% (53/4962), median ISS was 9 (IQR 9–17). 94·4% (*n* = 4733) of patients suffered blunt injuries, mainly comprising vehicle collisions (*n* = 2459, 49·6%) and falls under two metres (*n* = 1187, 23·9%). Penetrating trauma constituted only 4·6% (*n* = 229), mainly stabbing (2·6%, *n* = 130).

A comparison between included and excluded patients is shown in Table 4. Excluded patients had a comparable injury ISS (median 9 [IQR9–16] vs. median ISS 9 [IQR9–17], *p* < 0·01, respectively) and a higher mortality (3·1% vs. 1·1%, *p* < 0·01) relative to included patients. Excluded patients were more likely to have suffered burns and falls below two metres.

**Table 4:** Comparison of the characteristics of included versus excluded patients.

Variable	Included patients	Excluded patients
**Male gender, *n* (%)**	3447 (69·5%)*	6847 (67·3%)
**ISS, median (IQR)**	9 (9, 17)*	9 (9, 16)
**Age (years), median (IQR)**	11·9 (8·0, 14·2)*	3·9 (1·6, 10·1)
**Mortality, *n* (%)**	53 (1·1%)	316 (3·1%)*
**Injury type**		
Blunt	4733 (95·4%)	9935 (97·7%)*
Penetrating	229 (4·6%)*	236 (2·3%)
**Injury mechanism**		
Vehicle incident/collision	2459* (49·6%)	2262 (22·2%)
Fall less than 2m	1187* (23·9%)	4827 (47·5%)
Blow(s)	327 (6·6%)	943 (9·3%)
Crush	42 (0·9%)	118 (1·1%)
Fall more than 2m	645* (13·0%)	869 (8·5%)
Other	147 (3·0%)	971* (9·6%)
Burn	4 (0·1%)	40* (0·4%)
Stabbing	130* (2·6%)	99 (1·0%)
Shooting	16 (0·3%)	26 (0·3%)
Blast	5 (0·1%)	16 (0·2%)

Ledger: Patients were excluded on the basis of insufficient pre-hospital physiological data required to apply the tools (see Methods). OR=Odds ratio. Percentages represent the proportion of patients within the included (or excluded) group with the characteristic described (e.g. 69·5% of all included patients were of male gender). * indicates the group that has higher number of incidents than expected, and is statistically significant (*p* < 0·05).

### 3.2 Intervention-based designation of triage categories (using Lerner's criteria)

Fewer than 1% of patients (*n* = 31) met criteria for the “Dead” and “Expectant” category, with universal mortality across both groups (Table 5). 1343 patients fulfilled the criteria for P1 status: three quarters (*n* = 808) required advanced airway intervention, 28·9% (*n* = 310) arrived in ED with uncontrolled haemorrhage and 15·7% (*n* = 169) required time-critical major surgical intervention (Table 2). The remaining patients (*n* = 3588, 72·3%) were designated P2, representing the largest triage category. By virtue of TARN's inclusion criteria, no patients met criteria for the P3 (minor) category (Supplementary data Table 1).

**Table 5:** Comparison of outcome characteristics between patients in each triage category.

Triage category	Total, n (%)	Mortality, n (%)	ICU admission, n (%)	LOS (days), median (IQR)	ISS, median (IQR)
Dead	2 (0·04%)	2 (100·0%)	1 (50·0%)	11 [6, 15]	22 [21, 24]
Expectant	29 (0·58%)	29 (100·0%)	27 (93·1%)	1 [1, 3]	41 [29, 50]
Priority 1 (Immediate)	1343 (27·1%)	19 (1·4%)	809 (60·2%)	7 [3, 15]	17 [9, 26]
Priority 2 (Urgent)	3588 (72·3%)	3 (0·08%)	304 (8·5%)	5 [3, 9]	9 [9, 12]

Ledger: ISS=Injury Severity Score, IQR=interquartile range, LOS=Length of stay, ICU=Intensive Care Unit.

Patients assigned P1 based on Lerner's criteria suffered higher in-hospital mortality (1·41% vs*.* 0·08%, *p* < 0·01), had longer LOS (median 7 vs. 5 days, *p* < 0·01), suffered more severe injuries (median ISS 17[IQR 9–26] vs*.* 9[IQR9–12], *p* < 0·01), and were seven times more likely to require ICU admission (60·2% vs*.* 8·5%, *p* < 0·01) than patients designated P2.

### 3.3 Triage tool performance

Tool prediction of P1 status in all children <16 is shown in Table 6a. Overall, the BCD Triage Sieve demonstrated the highest sensitivity (75·8%), with an over-triage rate of 67·4%. The PTT had a much lower sensitivity at 44·8%, with over-triage of 45·5%. CareFlight and RAMP had very similar performance characteristics in <16 s, achieving the highest specificity (over 94%) and lowest over-triage rates (25·7% and 25·6%, respectively) of all the tools. MPTT and MPTT-24 exhibited the second highest sensitivity (59·2% and 56·7%, respectively), however these tools also exhibited the highest over-triage rates (74·8% and 74·3%). For comparison, tool performance in children <12 is shown in Table 6b.

**Table 6a:** Triage tool performance in predicting Priority 1 status in children ≤16 years (the need for time-critical major operative or resuscitative measures).

Age group	Tool	Sensitivity	Specificity	Under-triage	Over-triage	AUC
**All**	BCD Triage Sieve	75·7 (73·3, 78·0)	42·0 (40·3, 43·5)	24·3	67·4	0·588 (0·571, 0·606)
**(<16 years)**	CareFlight	40·4 (37·8, 43·1)	94·8 (94·0, 95·5)	59·6	25·7	0·676 (0·661, 0·692)
	JumpSTART	35·5 (33·0, 38·2)	93·5 (92·6, 94·3)	64·5	33·1	0·645 (0·629, 0·661)
	MIMMS Triage Sieve	37·5 (34·9, 40·2)	87·8 (86·7, 88·9)	62·5	46·7	0·627 (0·610, 0·643)
	MPTT	59·2 (56·5, 61·8)	34·8 (33·3, 36·4)	40·8	74·8	0·470 (0·452, 0·488)
	MPTT-24	56·7 (54·0, 59·3)	39·2 (37·6, 40·8)	43·3	74·3	0·479 (0·461, 0·497)
	MSTART	50·9 (48·2, 53·6)	88·0 (86·9, 89·0)	49·1	38·9	0·695 (0·679, 0·709)
	NARU Triage Sieve	48·3 (45·6, 51·0)	78·8 (77·4, 80·1)	51·7	54·2	0·636 (0·619, 0·652)
	PTT*	44·8 (40·9, 48·7)	87·2 (85·6, 88·7)	55·2	45·5	0·660 (0·637, 0·683)
	RAMP	39·7 (37·1, 42·4)	94·9 (94·2, 95·6)	60·3	25·6	0·673 (0·657, 0·689)
	START	49·2 (46·5, 51·9)	88·6 (87·6, 89·7)	50·8	38·3	0·689 (0·674, 0·705)

Ledger: BCD Triage Sieve =Battlefield Casualty Drills Triage Sieve, MIMMS Triage Sieve=Major Incident Medical Management System Triage Sieve, MPTT=Modified Physiological Triage Tool, MSTART=Modified START, NARU Triage Sieve=National Ambulance Resilience Unit Triage Sieve, RAMP=Rapid Assessment of Mentation and Pulse, START=Simple Triage and Rapid Treatment, PTT=Paediatric Triage Tape; *The PTT is only applicable to those under 12 years (*n* = 2516, 50·7%)·.

**Table 6b:** Triage tool performance in predicting Priority 1 status in children ≤12 years (the need for time-critical major operative or resuscitative measures).

Age group	Tool	Sensitivity	Specificity	Under-triage	Over-triage	AUC
**All**	BCD Triage Sieve	85·6 (82·6, 88·2)	28·3 (26·2, 30·4)	14·4	71	0·570 (0·545, 0·595)
**(<12 years)**	CareFlight	43·7 (39·8, 47·6)	95·5 (94·4, 96·3)	56·3	23·3	0·696 (0·674, 0·710)
	JumpSTART	35·6 (31·9, 39·4)	95·6 (94·6, 96·5)	64·4	26·5	0·656 (0·633, 0·679)
	MIMMS Triage Sieve	45·7 (41·8, 49·7)	82·4 (80·6, 84·1)	54·3	53	0·641 (0·617, 0·664)
	MPTT	66·5 (62·6, 70·1)	21·5 (19·7, 23·5)	33·5	77·5	0·440 (0·413, 0·466)
	MPTT-24	64·1 (60·3, 67·8)	26·2 (24·2, 28·3)	35·9	77·1	0·452 (0·425, 0·478)
	MSTART	59·8 (55·8, 63·6)	85·1 (83·4, 86·7)	40·2	42·1	0·724 (0·704, 0·745)
	NARU Triage Sieve	60·2 (56·3, 64·0)	70·4 (68·3, 72·4)	39·8	59	0·653 (0·630, 0·676)
	PTT	44·8 (40·9, 48·7)	87·2 (85·6, 88·7)	55·2	45·5	0·660 (0·637, 0·683)
	RAMP	43·7 (39·8, 47·6)	95·5 (94·4, 96·3)	56·3	23·3	0·696 (0·674, 0·718)
	START	59·6 (55·7, 63·4)	85·1 (83·4, 86·7)	40·4	42·2	0·724 (0·703, 0·744)

Ledger: BCD Triage Sieve =Battlefield Casualty Drills Triage Sieve, MIMMS Triage Sieve=Major Incident Medical Management System Triage Sieve, MPTT=Modified Physiological Triage Tool, MSTART=Modified START, NARU Triage Sieve=National Ambulance Resilience Unit Triage Sieve, RAMP=Rapid Assessment of Mentation and Pulse, START=Simple Triage and Rapid Treatment, PTT=Paediatric Triage Tape;.

Tool prediction of P1 status in children within each four yearly age subgroups is demonstrated in Table 7. The BCD Triage Sieve exhibited the highest sensitivity (66·7–90·2%) in all subgroups of children, demonstrating a 38·4–49·8% higher sensitivity in detecting P1 status in all subgroups of children <12 years as compared with the PTT. The BCD Triage Sieve exceeded the sensitivity of the NARU Triage Sieve in 12–16 year olds by 29·2% (sensitivity of 66·7% vs 37·5%). Amongst 12–16 years olds, CareFlight demonstrated identical sensitivity to the NARU Triage Sieve (37·5%) but with markedly lower over-triage (28·2% vs. 44·8%, respectively).

**Table 7:** Tool performance by age subgroup.

Age group	Tool	Sensitivity	Specificity	Under-triage	Over-triage	AUC
12–16 years*	BCD Triage Sieve	66·7 (63·0, 70·1)	56·7 (54·3, 59·0)	33·3	61·8	0·616 (0·593, 0·640)
	CareFlight	37·5 (33·9, 41·2)	94·1 (92·9, 95·1)	62·5	28·1	0·658 (0·635, 0·680)
	JumpSTART	35·5 (32·0, 39·2)	91·2 (89·7, 92·4)	64·5	38·2	0·633 (0·610, 0·656)
	MIMMS Triage Sieve	30·1 (26·7, 33·6)	93·6 (92·4, 94·7)	69·9	34·5	0·619 (0·595, 0·642)
	MPTT	52·6 (48·8, 56·3)	49·1 (46·7, 51·5)	47·4	70·6	0·508 (0·483, 0·534)
	MPTT-24	49·9 (46·1, 53·6)	53·2 (50·8, 55·5)	50·1	70·0	0·515 (0·490, 0·540)
	MSTART	42·9 (39·2, 46·6)	91·1 (89·6, 92·3)	57·1	34·1	0·670 (0·647, 0·692)
	NARU Triage Sieve	37·5 (33·9, 41·2)	87·8 (86·1, 89·3)	62·5	44·8	0·626 (0·603, 0·650)
	RAMP	36·0 (32·5, 39·7)	94·4 (93·2, 95·4)	64·0	27·9	0·652 (0·629, 0·675)
	START	39·7 (36·1, 43·5)	92·4 (91·1, 93·6)	60·3	32·1	0·661 (0·638, 0·683)
**8–12 years**	BCD Triage Sieve	82·6 (77·6, 86·7)	38·8 (35·8, 42·0)	17·4	72·0	0·607 (0·572, 0·642)
	CareFlight	44·6 (38·8, 50·6)	96·4 (95·0, 97·4)	55·4	22·0	0·705 (0·674, 0·736)
	JumpSTART	39·4 (33·7, 45·3)	95·5 (93·9, 96·6)	60·6	28·5	0·674 (0·642, 0·707)
	MIMMS Triage Sieve	44·2 (38·5, 50·2)	89·94(87·9, 91·7)	55·8	44·1	0·671 (0·638, 0·704)
	MPTT	61·0 (55·1, 66·6)	29·2 (26·4, 32·1)	39·0	80·1	0·451 (0·412, 0·489)
	MPTT-24	57·8 (51·9, 63·6)	35·9 (32·9, 39·0)	42·2	79·3	0·468 (0·431, 0·507)
	MSTART	58·9 (52·9, 64·6)	90·1 (88·1, 91·9)	41·1	36·7	0·745 (0·716, 0·774)
	NARU Triage Sieve	51·9 (45·9, 57·8)	81·5 (78·9, 83·8)	48·1	55·3	0·667 (0·634, 0·700)
	PTT	46·7 (40·8, 52·6)	90·4 (88·4, 92·2)	53·3	41·5	0·686 (0·654, 0·718)
	RAMP	44·6 (38·8, 50·6)	96·4 (95·0, 97·4)	55·4	22·0	0·705 (0·674, 0·736)
	START	58·9 (52·9, 64·6)	90·1 (88·1, 91·9)	41·1	36·7	0·745 (0·716, 0·774)
**4 −8 years**	BCD Triage Sieve	90·2 (85·0, 93·7)	14·8 (12·1, 18·1)	9·9	72·4	0·525 (0·479, 0·571)
	CareFlight	44·3 (37·4, 51·5)	97·2 (95·3, 98·3)	55·7	15·1	0·708 (0·669, 0·746)
	JumpSTART	35·5 (28·9, 42·5)	97·9 (96·2, 98·9)	64·5	14·3	0·667 (0·626, 0·707)
	MIMMS Triage Sieve	46·3 (39·3, 53·4)	81·6 (78·1, 84·6)	53·7	52·5	0·640 (0·597, 0·682)
	MPTT	72·9 (66·2, 78·8)	10·4 (8·1, 13·3)	27·1	77·4	0·417 (0·370, 0·464)
	MPTT-24	70·9 (64·1, 77·0)	12·9 (10·3, 16·0)	29·1	77·4	0·419 (0·373, 0·466)
	MSTART	61·1 (54·0, 67·8)	87·1 (84·0, 89·7)	38·9	37·1	0·741 (0·705, 0·777)
	NARU Triage Sieve	63·6 (56·6, 70·1)	65·5 (61·4, 69·4)	36·5	60·2	0·645 (0·603, 0·687)
	PTT	40·4 (33·7, 47·5)	91·0 (88·2, 93·2)	59·6	38·4	0·657 (0·616, 0·698)
	RAMP	44·3 (37·4, 51·5)	97·2 (95·3, 98·3)	55·7	15·1	0·708 (0·669, 0·746)
	START	60·6 (53·5, 67·3)	87·1 (84·0, 89·7)	39·4	37·2	0·738 (0·702, 0·775)
**0–4 years**	BCD Triage Sieve	85·4 (78·6, 90·5)	19·0 (14·9, 23·8)	14·6	66·5	0·522 (0·466, 0·578)
	CareFlight	41·1 (33·2, 49·4)	89·6 (85·5, 92·6)	58·9	34·7	0·653 (0·602, 0·704)
	JumpSTART	28·5 (21·6, 36·5)	92·1 (88·4, 94·7)	71·5	36·8	0·603 (0·550, 0·656)
	MIMMS Triage Sieve	47·7 (39·6, 55·9)	60·1 (54·5, 65·5)	52·3	63·6	0·539 (0·484, 0·594)
	MPTT	68·2 (60·1, 75·4)	17·4 (13·5, 22·1)	31·8	71·7	0·428 (0·372, 0·484)
	MPTT-24	66·9 (58·7, 74·2)	19·3 (15·2, 24·2)	33·1	71·6	0·431 (0·375, 0·487)
	MSTART	59·6 (51·3, 67·4)	65·8 (60·3, 71·0)	40·4	54·6	0·627 (0·575, 0·679)
	NARU Triage Sieve	71·5 (63·5, 78·4)	44·3 (38·8, 50·0)	28·5	62·0	0·579 (0·525, 0·633)
	PTT	47·0 (38·9, 55·3)	70·3 (64·8, 75·2)	53·0	57·0	0·586 (0·533, 0·640)
	RAMP	41·1 (33·2, 49·4)	89·6 (85·5, 92·6)	58·9	34·7	0·653 (0·602, 0·704)
	START	59·6 (51·3, 67·4)	65·8 (60·3, 71·0)	40·4	54·6	0·627 (0·575, 0·679)

Ledger: BCD Triage Sieve =Battlefield Casualty Drills Triage Sieve, MIMMS Triage Sieve=Major Incident Medical Management System Triage Sieve, MPTT=Modified Physiological Triage Tool, MSTART=Modified START, NARU Triage Sieve=National Ambulance Resilience Unit Triage Sieve, RAMP=Rapid Assessment of Mentation and Pulse, START=Simple Triage and Rapid Treatment, PTT=Paediatric Triage Tape. *The PTT is only applicable to those under 12 years (*n* = 2516, 50·7%).

CareFlight and RAMP exhibited very similar performance characteristics to each other as well as the most consistent performance across all age subgroups, with sensitivities in the region of 36·0 to 44·6% and specificity consistently ≥90%.

JumpSTART demonstrated low sensitivity in predicting P1 status in 4 to 8 year olds (35·5%) and 0 to 4 year olds (28·5%), accompanied by high specificity (over 90%) and therefore low over-triage rates (14·3% and 36·8%, respectively). However, START demonstrated nearly double the sensitivity (60.6% and 59.6%) in both these subgroups.

1617 of all patients <16 years had ISS>15; of these, only 52·5% (*n* = 849) met criteria for intervention-based P1 status. One third (*n* = 494) of intervention-based P1 patients had an ISS≤15. Amongst the tools, the BCD Triage Sieve exhibited the highest sensitivity in predicting mortality (94·3%) and ISS>15 (73·6%) (Supplementary data Tables 2 and 3).

## 4 Discussion

Care of children during MIs is challenging and emotive, and specialist paediatric trauma resources are less available than adult services. As such, the objective and accurate triage of children in MIs is vital to ensure that healthcare resources are appropriately allocated. This study has assessed the performance of 11 primary MI triage tools using data from 4962 injured children from the UK national TARN registry. The PTT, currently employed by UK ambulance services for use in children <12 years, correctly identified only 45% of children requiring time-critical major resuscitative and surgical interventions (P1 patients); whilst the highest sensitivity (75·7%) was demonstrated by the UK military adult tool, the BCD Triage Sieve. The US-based JumpSTART demonstrated low sensitivity in predicting P1 status in children <8 years (35·5% in 4 to 8 year olds and 28·5% in 0 to 4 year olds) and was outperformed by its adult counterpart START (60% sensitivity in children <8 years). Lerner's criteria with paediatric-specific fluid resuscitation measures have been used to define triage categories in a paediatric population, yielding clinically meaningful differences between patient groups.

Despite utilising age-specific paediatric respiratory and heart rate thresholds, the PTT is outperformed by the adult BCD Triage Sieve. This may be attributable to the BCD Triage Sieve's early application of the mental status assessment “Responds to voice?” (approximately equivalent to a GCS of 12) [26], as mental status correlates strongly with outcomes following trauma [11,12]. A US registry-based study examining triage tool performance, in which 36,618 out of 530,695 patients were aged <16 years, demonstrated that GCS was a strong predictor of mortality at hospital discharge in patients of all ages (AUC 0·825), particularly in children aged 0 to 8 years (AUC 0·964), where GCS outperformed CareFlight and START [11]. In our study, the BCD Triage Sieve has demonstrated twice the sensitivity (75·7% vs. 35·5%) in predicting the need for time-critical major resuscitative and/or surgical intervention (Priority 1 status) in injured children when compared with the currently utilised PTT, as well as enhanced sensitivity in predicting mortality (94·3%) and ISS>15 (73·6%). We recommend that the BCD Triage Sieve replace the PTT (and the NARU Triage Sieve used in children ≥12 years) as the primary MI triage tool for patients aged <16 years in the UK. The BCD Triage Sieve has a higher rate of over-triage compared to PTT (67·4% vs. 45·5%, respectively). This is comparable to the BCD Triage Sieve's 70·9% over-triage rate demonstrated in an adult study [18], which demonstrated the BCD Triage Sieve offers optimal performance in predicting P1 status in adults [18]. Having one primary MI triage tool for use across all ages would simplify EMS training and improve the consistency of triage. Human factors affect performance during MIs [8], and in incidents involving an ongoing threat, first responders trained in triage methods that employ arithmetic often resort to more simplistic means, as noted following the San Bernadino shootings [3]. Avoiding tools that involve the application of more complex, age-specific physiological parameters (e.g. PTT) in the triage of children is likely to be associated with more reliable triage in practice. Care providers may also choose to employ the BCD Triage Sieve in casualty clearing stations and at hospital reception in the absence of an effective secondary paediatric MI triage tool.

Our study demonstrated that CareFlight and PTT had similar sensitivity in predicting P1 status (40·4% and 44·7%, respectively), consistent with a prospective South African study of 3461 children (<13 years) presenting to ED, which demonstrated that CareFlight and PTT had comparable sensitivity (46% and 41·5%, respectively) in predicting the need for urgent non-orthopaedic surgery or other resuscitative intervention [19]. This study, similar to ours, also highlighted that JumpSTART had the lowest sensitivity (0·8%) of all tools tested: JumpSTART is intended to replace START in children <8 years; however, in our study it is outperformed by START in all age subgroups. Based on this and prior evidence, regions employing JumpSTART may wish to consider alternative methods to triage children in MIs.

A key strength of this study is use of Lerner's criteria (expanded to include paediatric-specific fluid resuscitation measures) to define triage categories, which has several advantages over using ISS>15 or intervention-based criteria described previously [12,22]. ISS>15 is widely used in quality assurance as the threshold to justify the highest tier of trauma care in the UK and US: however ISS correlates poorly with the need for medical intervention, as our study confirms[23]. In 2001, Garner described criteria for defining Priority 1 status, including non-orthopaedic surgery within 6 h and other resuscitative measures [12]. Garner included in their definition of P1 patients who received over 1000 ml of fluid to maintain a blood pressure above 89 mmHg (which is less common in current practice, with preferential use of blood and blood products), and those undergoing invasive intracranial pressure monitoring, which has since been shown to lack correlation with neurological outcome. In 2006, Wallis described triage categories (P1, P2 and P3 equivalents) derived using a Delphi consensus of experts, however, these were only applicable to children and outlined aggressive time cut-offs more akin to combat casualty care (e.g. P1 casualties are those requiring laparotomy or thoracotomy within one hour) [22]. By comparison, our study has utilised Lerner's criteria (derived by expert consensus and literature review), rather than author-defined criteria alone [9]. Lerner's system has multiple advantages: it defines all possible triage categories, it considers a broad range of injury mechanisms including burns, its use has been validated in adults [18] and it is applicable to patients of all ages (allowing children and adults to be considered simultaneously), which may facilitate more equitable resource allocation [9]. Furthermore, we have demonstrated clinically meaningful differences in mortality, ISS and ICU requirement in patient groups constituting each triage category. In a previous Utstein-style consensus on the reporting of the acute medical response to disasters, experts highlighted the need to define a universally accepted measure of triage accuracy, particularly to establish whether criteria used to sort injured survivors into categories are clinically meaningful and are adequately predictive of survivability [32]. We recommend use of Lerner's definitions of triage categories (with paediatric-specific fluid resuscitation measures, where applicable) as an objective, evidence-based means by which to model novel tools and to define triage categories when conducting post-event evaluations of UK and international MI triage**.** Uniformity in reporting of MI triage will allow meaningful comparison between studies and thereby facilitate refinement in MI policy [4].

Other study strengths include use of trauma registry data, allowing tool performance to be assessed on a large, nationally representative sample of injured children, incorporating multiple injury mechanisms. This overcomes the practical limitations of conducting studies during actual MIs. Computed application of triage tools has allowed the inherent discriminatory capability of triage tools to be assessed independently of human error.

Study limitations include under-representation of burns and blast injury mechanisms. The low proportion of patients with gunshot wounds is representative of the UK, where mass shootings are rare following the introduction of strict gun laws after the 1996 Dunblane Massacre [4,6]. Our study findings may be less generalisable to other nations and during conflict [4]. The term “catastrophic haemorrhage” utilised by four tools could not be applied using registry information; however, there is abundant evidence that haemorrhage is the leading preventable cause of death following trauma and that control of bleeding improves survival [33]. This study focusses on the ability of triage tools to predict P1 status only. Future studies should evaluate the ability of tools to predict other triage categories (e.g. over-triage of P3 patients as P2 may impact hospital resources) and further reduce over-triage rates. Over-triage has the advantage of rapidly removing children from the scene; however, there is a direct correlation between over-triage and mortality [7]. Further work should focus on developing tools that do not involve arithmetic calculation: CareFlight and RAMP employ qualitative assessments alone, however, both have demonstrated sensitivity <50% in predicting P1 status in this and other studies. Our study findings may be biased by patients excluded due to missing pre-hospital physiological data: excluded patients had a higher mortality and younger age when compared with included patients. In particular, our study's estimation of tools’ ability to predict mortality as an outcome measure is likely further biased by TARN's exclusion of pre-hospital deaths. It is unclear why such a large proportion of children (67·2%) within the trauma registry are missing pre-hospital data as compared with 9·2% of adults in a similar study [18]. Possible explanations include challenges in collecting prehospital observations in young children, expedited transfer of paediatric casualties to hospital or shortfalls in submitting data to TARN. We strongly recommend that care providers explore and address why the quality of paediatric prehospital data is remarkably different from that of adults within the same trauma registry. Several post-event evaluations have cited the availability of pre-hospital data as a barrier to determining MI triage tool performance [3,4,32]. We considered data imputation and use of first recorded hospital physiology (which may be influenced by treatments administered prior to hospital arrival), however these may further bias results. Although not without limitation, use of national trauma registry data may represent the largest UK population of injured children in whom triage tool performance can be assessed.

In conclusion, based on performance assessed using this trauma registry population, we recommend that the BCD Triage Sieve should be applied to both children and adults injured in UK MIs, which would simplify both training and application of the triage process while improving in parallel the accuracy in identifying patients in need of time-critical major resuscitative and surgical intervention. The methodology used in this study (Lerner's criteria, incorporating paediatric-specific fluid resuscitation measures) uses outcome data to identify appropriateness of original triage category. This method provides an objective standard for developing novel triage tools as well as conducting post-event evaluations of future UK MIs.

## 5 Author contributions

NM conducted a literature review prior to the study. NM, DB, DK and GVG designed the study. NM, SC and YX accessed the database, verified the underlying data and conducted analysis. All authors contributed to data interpretation. NM wrote the initial draft of the manuscript. All authors contributed to critical revisions of subsequent manuscript drafts and approve of the final version.

## Declaration of Competing Interest

The authors confirm that they have no conflicts of interest to declare.

## Appendix Supplementary materials

Image, application 1

Image, application 2
