# Supplementary material for: Paediatric major incident triage: UK military tool offers best performance in predicting the need for time-critical major surgical and resuscitative intervention
Source: eClinicalMedicine. 2021 Aug 23;40:101100. doi: 10.1016/j.eclinm.2021.101100 (PMC8548919; doi:10.1016/j.eclinm.2021.101100)
Supplement: Supplementary file 1 [file mmc1.docx]

**Supplementary Data Table 1: Designation of Triage Categories based on EMS and Hospital Interventions received:**

|  | **DEAD** | **TARN equivalent** |
| --- | --- | --- |
| 1· | A lack of palpable pulse and/or respiratory effort (i·e· cardiac or respiratory arrest) at initial EMS assessment that is not responsive to airway positioning or needle decompression | Any cardiorespiratory resuscitation at scene AND Inpatient death |
| 2· | Lack of pulse or respiratory effort within 15 minutes of EMS arrival at scene | Any cardiorespiratory resuscitation at scene AND Inpatient death |
| 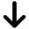**All no** | | |
|  | **EXPECTANT** | **TARN equivalent** |
| 1· | In patients aged 0 to 49 years old, third degree (full thickness) burns to >90% of the body | 90% Total Body Surface Burns |
| 2· | In patients over 50 years old, third degree (full thickness) burns to >80% of the body | Age≥50 years AND 40-89% Total Body Surface Burns |
| 3· | Penetrating or blunt trauma to the head which crosses the midline with agonal respirations and/or no motor response decorticate posturing or decerebrate posturing (i·e· a motor GCS of 3 or less) | Any head injury AND Total GCS=3 or Motor GCS≤3 at scene AND Inpatient death |
| 4· | Uncontrolled haemorrhage that resulted in cardiac arrest prior to EMS transport | *No TARN equivalent* |
| 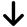**All no** | | |
|  | **PRIORITY 1 (IMMEDIATE)** | **TARN equivalent** |
| 1· | Neurologic, vascular, or haemorrhage-controlling surgery to the head, neck or torso performed within 4 hours of arrival to hospital | At scene: Pericardial decompression, pericardiocentesis, thoracotomy, heart surgery  In hospital (including ED):  Evacuation of EDH/SDH, Evacuation ICH, Elevation depressed cranium, Repair Cranium fracture, Open Craniotomy, Burrhole of Cranium, Lobectomy of brain, Repair of Dura, Craniectomy  Laparotomy, Splenectomy, Nephrectomy, Resection Liver, Repair Spleen, Repair Kidney laceration, Abdominal Packing, Caesarian Delivery for trauma, Colostomy, Hemicolectomy/Colectomy, Ileectomy, Repair Liver laceration, Repair Colon laceration, Repair Rupture to Bladder, Repair mesentery of small bowel, Repair mesentery of colon, Excision of Pancreas, Repair of Duodenum, Repair of Jejunum, Repair of Ileum, Repair of Stomach, Rectal operation, Bowel operations (specified),  Surgery involving the Iliac artery, Surgery involving the Subclavian artery, Aortic Repair, Pericardiocentesis, Thoracotomy, Aortic Repair, Pneumonectomy, Heart Surgery, Repair of lung, Repair Oesophagus, Diaphragm repair  External Fixation of Pelvis, Fixation of Pelvic Ring, Fixation of Acetabulum |
| 2· | Limb-conserving surgery performed within 4 hours of arrival at hospital on a limb that was found to be pulseless distal to the injury prior to surgery | Fasciotomy, Surgery to the brachial or femoral artery, Amputation of upper/lower limb·  Any injury to the brachial or femoral artery (not time dependent) |
| 3· | Escharotomy performed on a patient with burns within 2 hours of arrival at a hospital | Escharotomy |
| 4· | Chest tube placed within 2 hours of arrival at hospital | Insertion of chest tube at scene or in-hospital |
| 5· | An advanced airway intervention (e·g· intubation, LMA, surgical airway) performed in the pre-hospital setting or within 4 hours of arrival at hospital | Airway obstruction at scene or in-hospital,  Airway support required at scene or in-hospital,  Intubation and mechanical ventilation at scene or in-hospital,  CPAP administration in ED or Critical Care,  Cricothyroidotomy or tracheostomy required at scene or in-hospital |
| 6· | IV vasopressors administered within 2 hours of arrival at hospital | Administration of vasopressors/inotropes |
| 7· | Arrived in the ED with uncontrolled haemorrhage | Administration of 4 or more units of blood products within 24 hours of admission,  Any use of Resuscitative Endovascular Balloon Occlusion of the Aorta (REBOA), Interventional radiology/embolisation within 4 hours of hospital arrival  ***Administration of blood and/or blood products within 1 hour of hospital arrival**  ***Administration of >20ml/kg of intravenous fluids within 1 hour of hospital arrival** |
| 9· | Patient who required EMS initiation of CPR (i·e· had a cardiac arrest) during transport, in the ED, or within 4 hours of arrival at a hospital | Any cardiopulmonary resuscitation in hospital (in the absence of cardiopulmonary resuscitation at scene) |
| 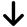**All no** | | |
|  | **PRIORITY 3 (MINIMAL)** | **TARN equivalent** |
| 1· | Discharged from the ED with no X-rays or an extremity X-ray that was negative or showed an uncomplicated fracture (i·e· a closed extremity fracture without significant displacement or neurovascular compromise); no laboratory testing; received only simple wound repair (single layer suturing only); and received no medications intravenously (does not include fluids), or inhaled (does not include oxygen) from EMS or in the hospital | *Not included in TARN database (these patients would not meet TARN inclusion criteria)* |
| **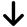Any no** | | |
|  | **PRIORITY 2 (DELAYED)** | **TARN equivalent** |
| 1· | All remaining patients | All remaining TARN patients who do not fulfil any criteria for previous triage categories |

Ledger: *These parameters are specific to children (aged <16years). Cardiac arrest is defined as a lack of palpable pulse and EMS initiation of CPR. CPR= cardiopulmonary resuscitation. Chemical, biological, radiological and neurological (CBRN) entities were excluded from triage category definitions as no CBRN patients exist within the TARN database. There is international variation in the names of triage categories with approximate equivalence as follows: Priority 1 (Immediate, Red, T1); Priority 2 (Delayed, T2, Urgent); Priority 3 (Minimal, Minor, T3); Expectant (Unsalvageable, T1 hold, P4); Dead (Black).

**Supplementary Table 2: Tool prediction of in-hospital mortality (patients under 16 years)**

| **Tool** | **Sensitivity** | **Specificity** | **Under-triage** | **Over-triage** | **AUC** |
| --- | --- | --- | --- | --- | --- |
| BCD Triage Sieve | 94·3 (83·4, 98·5) | 37·5 (36·1, 38·9) | 5·7 | 98·4 | 0·659 (0·594, 0·724) |
| CareFlight | 83·0 (69·7, 91·5) | 86·0 (85·0, 87·0) | 17·0 | 94·0 | 0·845 (0·809, 0·881) |
| JumpSTART | 86·8 (74·0, 94·1) | 86·4 (85·4, 87·4) | 13·2 | 93·5 | 0·866 (0·834, 0·898) |
| MIMMS Triage Sieve | 88·7 (76·3, 95·3) | 81·7 (80·6, 82·8) | 11·3 | 95·0 | 0·852 (0·817, 0·887) |
| MPTT | 18·9 (9·9, 32·4) | 36·0 (34·6, 37·3) | 81·1 | 99·7 | 0·274 (0·196, 0·352) |
| MPTT-24 | 17·0 (8·5, 30·3) | 39·8 (38·5, 41·2) | 83·0 | 99·7 | 0·284 (0·205, 0·363) |
| MSTART | 92·5 (80·9, 97·6) | 78·2 (77·0, 79·3) | 7·5 | 95·6 | 0·853 (0·819, 0·888) |
| NARU Triage Sieve | 84·9 (71·9, 92·8) | 72·1 (70·8, 73·3) | 15·1 | 96·8 | 0·785 (0·738, 0·832) |
| PTT* | 69·8 (55·5, 81·3) | 86·2 (85·2, 87·1) | 30·2 | 94·8 | 0·780 (0·732, 0·828) |
| RAMP | 90·6 (78·6, 96·5) | 79·1 (78·0, 80·3) | 9·4 | 95·5 | 0·849 (0·813, 0·884) |
| START | 91·7 (71·5, 98·5) | 79·7 (78·1, 81·3) | 8·3 | 95·8 | 0·801 (0·807, 0·907) |

Ledger: BCD Triage Sieve =Battlefield Casualty Drills Triage Sieve, MIMMS Triage Sieve=Major Incident Medical Management System Triage Sieve, MPTT=Modified Physiological Triage Tool, MSTART=Modified START, NARU Triage Sieve=National Ambulance Resilience Unit Triage Sieve, RAMP=Rapid Assessment of Mentation and Pulse, START=Simple Triage and Rapid Treatment, PTT=Paediatric Triage Tape. *The PTT is only applicable to those under 12 years (n=2516, 50·7%).

**Supplementary Table 3: Tool prediction of ISS>15**

| **Tool** | **Sensitivity** | **Specificity** | **Under-triage** | **Over-triage** | **AUC** |
| --- | --- | --- | --- | --- | --- |
| BCD Triage Sieve | 73·6 (71·4, 75·7) | 42·4 (40·7, 44·1) | 26·4 | 61·8 | 0·580 (0·563, 0·596) |
| CareFlight | 35·3 (33·0, 37·7) | 95·2 (94·4, 95·9) | 64·7 | 21·9 | 0·653 (0·637, 0·668) |
| JumpSTART | 29·9 (27·6, 32·2) | 93·1 (92·2, 94·0) | 70·1 | 32·3 | 0·615 (0·599, 0·631) |
| MIMMS Triage Sieve | 32·8 (30·5, 35·1) | 87·6 (86·4, 88·7) | 67·2 | 43·9 | 0·602 (0·586, 0·618) |
| MPTT | 61·7 (59·3, 64·1) | 35·6 (33·9, 37·2) | 38·3 | 68·4 | 0·486 (0·469, 0·504) |
| MPTT-24 | 59·4 (57·0, 61·8) | 40·2 (38·5, 41·8) | 40·6 | 67·6 | 0·498 (0·481, 0·515) |
| MSTART | 43·5 (41·1, 46·0) | 87·6 (86·4, 88·7) | 56·5 | 37·1 | 0·656 (0·640, 0·671) |
| NARU Triage Sieve | 43·1 (40·7, 45·6) | 78·5 (77·0, 79·9) | 56·9 | 50·8 | 0·608 (0·592, 0·624) |
| PTT* | 34·5 (32·2, 36·9) | 95·3 (94·5, 96·0) | 65·5 | 22·1 | 0·649 (0·633, 0·665) |
| RAMP | 42·4 (40·0, 44·8) | 88·4 (87·3, 89·5) | 57·6 | 36·1 | 0·654 (0·638, 0·670) |
| START | 36·4 (33·0, 40·0) | 85·6 (83·8, 87·2) | 63·6 | 48·2 | 0·610 (0·587, 0·633) |

Ledger: BCD Triage Sieve =Battlefield Casualty Drills Triage Sieve, MIMMS Triage Sieve=Major Incident Medical Management System Triage Sieve, MPTT=Modified Physiological Triage Tool, MSTART=Modified START, NARU Triage Sieve=National Ambulance Resilience Unit Triage Sieve, RAMP=Rapid Assessment of Mentation and Pulse, START=Simple Triage and Rapid Treatment, PTT=Paediatric Triage Tape. *The PTT is only applicable to those under 12 years (n=2516, 50·7%).

**Supplementary data: Analysis of missing data (relationship with patient characteristics/outcome and age)**

Number of patients with missing data:

Respiratory rate (ASSESS_RESP_RATE_VAL) 7477

Heart rate (ASSESS_PULSE_VAL) 7194

Total GCS Score (ASSESS_GCS_TOTAL) 6887

Systolic Blood Pressure (ASSESS_SYSBP_VAL) 9534

GCS Motor Score (ASSESS_GCS_MOTOR) 7275


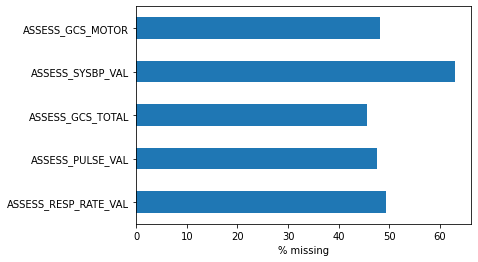


We examined whether missing physiology has any association with patients’ characteristics i.e. gender, injury type, injury mechanism, outcome, and age.

- There is an association between missing systolic blood pressure and patient gender (p = 0.0129). Females were more likely to have missing BP.
- There are associations between missing physiology and injury type (P < 0.0001). Patients with blunt injury were more likely to have missing HR, GCS TOTAL, GCS MOTOR, and BP. Patients with penetrating injury were more likely to have missing RR.
- There is an association between missing physiological data and outcome (p < 0.01). Patients with missing RR, HR, GCS TOTAL, GCS MOTOR were less likely to die. Patients with missing BP were more likely to die.
- There is an association between missing physiological data and INJURY MECHANISM (p < 0.01). Patients with missing RR, HR, GCS TOTAL and MOTOR were more likely to have the following injury: blow, fall less than 2M, and other. Patients with missing BP were also more likely to have burn injury.
- Patients with missing physiological data tend to be younger (median age 3.6 *vs*. 9.9 years old). The following graph demonstrates the relationship between age and missing physiological data.


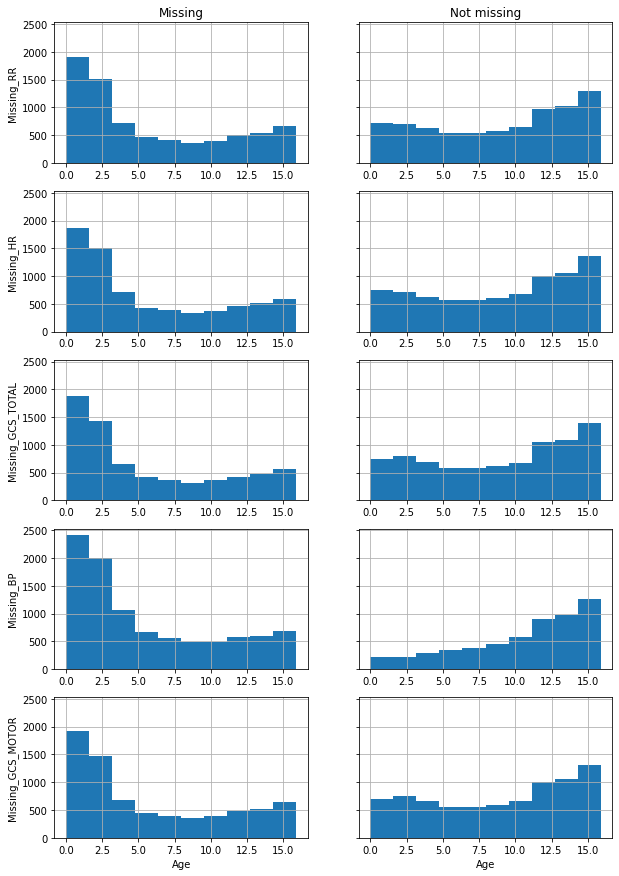


Ledger: GCS=Glasgow Coma Score, BP=systolic blood pressure, HR=heart rate, RR=respiratory rate
