# Supplementary material for: Paediatric major incident triage: UK military tool offers best performance in predicting the need for time-critical major surgical and resuscitative intervention
Source: eClinicalMedicine. 2021 Aug 23;40:101100. doi: 10.1016/j.eclinm.2021.101100 (PMC8548919; doi:10.1016/j.eclinm.2021.101100)
Supplement: Supplementary file 2 [file mmc2.docx]

**Research in context**

Evidence before this study

Children are frequently injured during major incidents, including natural disasters and terrorist attacks. When the immediate needs of the injured exceed the resources available to treat them, accurate triage is vital to ensure that healthcare resources are allocated to maximise overall survival. The additional emotional burden in triaging injured children confounds objective and accurate triage. Several major incident triage tools have been described internationally, however there are challenges in drawing meaningful conclusions about their effectiveness, due to differences in study context, patient populations and outcome measures.

Added value of this study

This study interrogates the UK national Trauma Audit and Research Network registry to assess the ability of nine adult and two dedicated paediatric triage tools to predict the need for time-critical major resuscitative and surgical intervention (Priority 1 (P1) status) in 4,962 injured children aged <16 years. Intervention-based definitions of triage categories (Lerner’s criteria) have been applied in children.

The UK military’s Battlefield Casualty Drills (BCD) Triage Sieve demonstrated the greatest sensitivity (75.8%) in predicting P1 status, outperforming both the UK Paediatric Triage Tape (PTT, currently used by the UK national Ambulance Service for children <12 years) and the National Ambulance Resilience Unit (NARU) Triage Sieve (currently used for 12-16 year olds) by 30%. Unexpectedly, JumpSTART (designed for children <8 years) was markedly outperformed by its adult counterpart START.

Implications of all the available evidence

Various studies have demonstrated that the PTT has a sensitivity of <50% in predicting the need for life-saving intervention in injured children. The BCD Triage Sieve may deliver a 30% improvement in performance, which is likely to improve overall survival following UK major incidents. Several studies have highlighted the suboptimal performance of JumpSTART; regions employing this tool may wish to consider use of alternative triage tools.

The methodology applied in this study (Lerner’s criteria with paediatric-specific fluid resuscitation measures) uses outcome data to identify appropriateness of original triage category. This method provides an objective standard for developing novel triage tools in children as well as conducting post-event evaluations of future UK major incidents.
